# Octamer-binding factor 6 (Oct-6/Pou3f1) is induced by interferon and contributes to dsRNA-mediated transcriptional responses

**DOI:** 10.1186/1471-2121-11-61

**Published:** 2010-08-05

**Authors:** Elisabeth Hofmann, Ursula Reichart, Christian Gausterer, Christian Guelly, Dies Meijer, Mathias Müller, Birgit Strobl

**Affiliations:** 1 Institute of Animal Breeding and Genetics, University of Veterinary Medicine Vienna, Vienna, Austria; 2 Center for Medical Research, Medical University of Graz, Graz, Austria; 3 Department of Cell Biology and Genetics, ErasmusMC, Rotterdam, Netherlands; 4 Biomodels Austria, University of Veterinary Medicine Vienna, Vienna, Austria; 5 Department of Forensic Medicine, Medical University of Vienna, Austria

## Abstract

**Background:**

Octamer-binding factor 6 (Oct-6, Pou3f1, SCIP, Tst-1) is a transcription factor of the Pit-Oct-Unc (POU) family. POU proteins regulate key developmental processes and have been identified from a diverse range of species. Oct-6 expression is described to be confined to the developing brain, Schwann cells, oligodendrocyte precursors, testes, and skin. Its function is primarily characterised in Schwann cells, where it is required for correctly timed transition to the myelinating state. In the present study, we report that Oct-6 is an interferon (IFN)-inducible protein and show for the first time expression in murine fibroblasts and macrophages.

**Results:**

Oct-6 was induced by type I and type II IFN, but not by interleukin-6. Induction of Oct-6 after IFNβ treatment was mainly dependent on signal transducer and activator of transcription 1 (Stat1) and partially on tyrosine kinase 2 (Tyk2). Chromatin immunopreciptitation experiments revealed binding of Stat1 to the Oct-6 promoter in a region around 500 bp upstream of the transcription start site, a region different from the downstream regulatory element involved in Schwann cell-specific Oct-6 expression. Oct-6 was also induced by dsRNA treatment and during viral infections, in both cases *via *autocrine/paracrine actions of IFNα/β. Using microarray and RT-qPCR, we furthermore show that Oct-6 is involved in the regulation of transcriptional responses to dsRNA, in particular in the gene regulation of serine/threonine protein kinase 40 (*Stk40*) and U7 snRNA-associated Sm-like protein Lsm10 (*Lsm10)*.

**Conclusion:**

Our data show that Oct-6 expression is not as restricted as previously assumed. Induction of Oct-6 by IFNs and viruses in at least two different cell types, and involvement of Oct-6 in gene regulation after dsRNA treatment, suggest novel functions of Oct-6 in innate immune responses.

## Backgound

Type I interferons (IFNα/β) are pleiotropic cytokines that exhibit antiviral, antiproliferative and immunomodulatory effects [1,2]. IFNα/β signal through the Janus kinase (Jak)/signal transducer and activator of transcription (Stat) cascade [3,4]. Upon binding of IFNα/β to its cognate receptor (consisting of Ifnar1 and Ifnar2) a series of phosphorylation events exerted by the associated kinases Jak1 and Tyk2 leads to the activation and nuclear translocation of mainly Stat1/Stat2 heterodimers. Stat1/Stat2 together with IFN regulatory factor 9 (Irf9) form the transcription factor complex IFN-stimulated gene factor 3 (ISGF3), which binds to promoters containing an interferon stimulated response element (ISRE, [5]). To a lesser extent Stat1 homodimers are activated and induce the expression of genes containing an IFNγ activated site (GAS, [5]) in their promoter. Type II IFN (IFNγ) activates mainly Stat1 homodimers, low levels of ISGF3 [6,7] and induces an overlapping but not identical set of genes as type I IFNs [4,8]. Additional Stats (e.g. Stat3, Stat5) may also be activated by both type I and type II IFNs in a more cell type-restricted manner, but their contribution to IFN-triggered responses is less well established [9]. In addition to the so-called canonical Jak/Stat pathway, other signalling cascades can be activated and impact on gene regulation [4]. Hundreds of IFN stimulated genes (ISGs), many of them still poorly characterised [10,11], mediate the complex biological responses to IFNs.

Oct-6 (Pou3f1, SCIP, Tst-1) is a member of the Pit-Oct-Unc (POU) family of transcription factors [12,13]. These proteins are characterised by the highly conserved structure of their DNA-binding domain, the POU-domain, consisting of a POU-specific domain and a POU-homeodomain. Consequently, POU-domain transcription factors recognise a common motif, the octamer consensus motif (ATGCAAAT; [12]). Members of this family are involved in a variety of cellular processes, ranging from house-keeping gene function (Oct-1) to programming of embryonic stem cells (Oct-4), development of the immune system (Oct-1 and Oct-2), of the pituitary gland (Pit-1) or of the nervous system (Brn-1 through -4 and Oct-6). Oct-6 belongs to the POU protein class III family, whose members are mainly involved in neuronal development [13]. Oct-6 expression is considered cell type-restricted and has so far been described in embryonic stem cells [14,15], developing neural and glial cells [16], cells of neonatal testes [17], squamous epithelia [18], proliferating epidermal keratinocytes [19], and pancreatic β-cells [20]. Oct-6 function is mainly characterised in Schwann cells [21,22] and considerably less is known about its role in other cell types. Oct-6 is crucial for the terminal differentiation of myelinating Schwann cells and is required for the expression of early growth response protein 2 (Egr2/Krox20), another transcription factor critical for Schwann cell development [23,24]. Oct-6-deficient mice display severe defects in peripheral nerve myelination and, in addition, die soon after birth from a breathing insufficiency caused by defective migration and differentiation of certain neurons in the brainstem [21,22]. Mice with a Schwann cell-specific Oct-6 knockout (i.e. deletion of the Schwann cell-specific enhancer element, SCE) are viable and have severe myelination defects in the peripheral nervous system, as Schwann cells are transiently arrested in the promyelinating stage [25]. Mice expressing Oct-6 constitutively in Schwann cells show a persistent block in myelination [26]. These mice have normal levels of *Egr2*, but the levels of myelin genes such as myelin protein zero (*Mpz*), myelin basic protein (*Mbp*), and peripheral myelin protein 22 (*Pmp22*) are significantly reduced [26]. Thus Oct-6 exerts activating as well as repressing functions during myelination and correctly timed, transient expression of Oct-6 is crucial for the development of myelinating Schwann cells. Although Oct-6 is also expressed in oligodendrocytes, Oct-6-deficient mice do not show any myelination defects in the central nervous system, supposedly due to functional redundancies with other co-expressed members of the POU protein class III family [27]. However, transgenic overexpression of Oct-6 in oligodendrocytes causes defective myelination and severe neurological disease, arguing for an impact of at least de-regulated Oct-6 on central nervous system myelination [28].

Here, we show for the first time cytokine inducibility of Oct-6 and its expression in cell types other than those described before. Oct-6 expression was observed in fibroblasts and macrophages in response to type I and type II IFN, during viral infections, and after treatment with the dsRNA analogue poly(I:C). We demonstrate that the IFNβ-mediated induction is Stat1-dependent and we identify a Stat1-binding region in the *Oct-6 *promoter. In addition, we compared the transcriptomes of wild type (WT) and Oct-6-deficient macrophages and show an involvement of Oct-6 in the transcriptional control of a subset of poly(I:C) responsive genes.

## Results

### Oct-6 is induced by IFNβ treatment in fibroblasts and in a Schwann cell line

**Figure 1:**
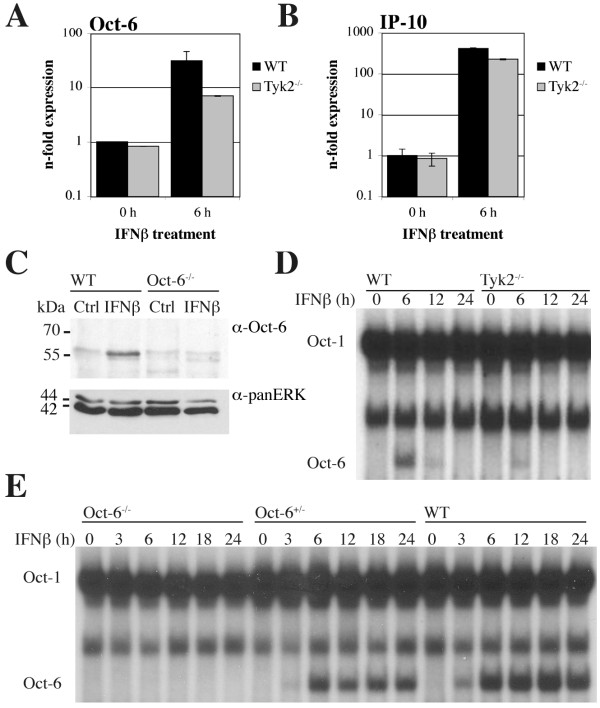
***Oct-6 *mRNA and protein is induced by IFNβ in primary fibroblasts (pMEFs) in a Tyk2-dependent manner**. WT and Tyk2^-/- ^(A, B) pMEFs were treated with IFNβ for 6 h or incubated with medium alone (Ctrl). (A) *Oct-6 *and (B) *IP-10 *mRNA levels were determined by RT-qPCR using *Ube2d2 *as endogenous control and calculated relative to untreated WT cells. Mean values ± SD of two independent experiments are shown. (C) WT and Oct-6^-/- ^pMEFs were treated with IFNβ for 6 h or incubated with medium alone (0 h). Oct-6 was immunoprecipitated from whole cell extracts; panERK was used as an input control. (D) WT and Tyk2^-/- ^pMEFs were treated with IFNβ for the times indicated. Whole cell extracts were analysed by EMSA with an octamer motif containing oligonucleotide. (E) Oct6^-/-^, Oct6^+/- ^and WT pMEFs were treated with IFNβ for the times indicated. Whole cell extracts were analysed by EMSA as described in (D). (C - E) Representatives of at least two independent experiments are shown.

Expression profiling of Tyk2-deficient fibroblast cell lines revealed that *Oct-6 *is a gene being strongly induced by IFNβ and that depends on the presence of Tyk2 for its full expression (C. Gausterer, B. Strobl et al., unpublished). Since neither IFN inducibility nor expression of *Oct-6 *in fibroblasts has been described before, we confirmed data in primary embryonic fibroblasts (pMEF) using RT-qPCR analysis. *Oct-6 *mRNA expression was clearly induced by IFNβ treatment and induction was reduced in the absence of Tyk2 (Figure 1A). Induction of *Oct-6 *was lower than observed for the known IFN target gene C-X-C motif chemokine 10 (*Cxcl10/IP-10*, Figure 1B), one of the genes most strongly induced by IFNs. In order to test if Oct-6 expression in fibroblasts is also detectable at the protein level, we performed immunoprecipitation experiments. Oct-6 protein was clearly induced by IFNβ treatment (Figure 1C). In untreated cells, Oct-6 protein could not be detected, a background band appeared at the same intensity and similar molecular weight as in Oct-6-deficient cells. We next tested Oct-6 DNA-binding activity with EMSAs using an oligonucleotide that contains an octamer consensus motif. As shown in Figure 1D, three DNA-binding complexes were found in IFNβ treated pMEFs. The fastest migrating complex was only observed after treatment with IFNβ and was hardly detectable in Tyk2-deficient cells. This complex was identified as Oct-6 by supershift with a specific antibody (see additional file 1) and by its absence in Oct-6^-/- ^cells after IFNβ treatment (Figure 1E). The two other complexes were present in all samples irrespective of genotype and treatment. The slowest migrating complex was identified as the ubiquitously expressed Oct-1, the other one could so far not be assigned to a specific octamer-binding protein and did not supershift with Oct-1, Oct-2 or Oct-6 specific antibodies (see additional file 1). Of note, duration of Oct-6 expression in response to IFNβ appeared consistently different in cells derived from mice with distinct genetic backgrounds (compare WT in Figure 1D and Figure 1E).

**Figure 2:**
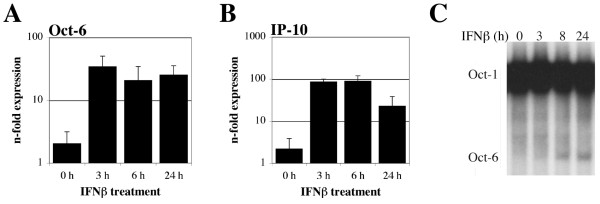
**IFNβ induces expression of Oct-6 in the murine Schwann cell line SW10**. Cells were treated with IFNβ for the times indicated or incubated with medium alone (0 h). (A) *Oct-6 *and (B) *IP-10 *mRNA levels were determined by RT-qPCR as described in the legend to Figure 1B. Data are depicted relative to cells incubated without IFNβ from one of the experiments. Mean values ± SD of two independent experiments are shown. (C) Whole cell extracts were analysed by EMSA as described in the legend of Figure 1 D. A representative of two independent experiments is shown.

Since Oct-6 expression and function is best characterised in developing Schwann cells, we analysed whether IFNβ can induce Oct-6 expression in this cell type. IFNβ treatment of the murine Schwann cell line SW10 resulted in a rapid and clear increase of *Oct-6 *and *IP-10 *mRNAs (Figure 2A and 2B). Oct-6 protein was detectable by EMSA at around 3 h to 8 h after IFNβ stimulation, although levels were consistently quite low (Figure 2C). In accordance with their immature phenotype [29], Oct-6 DNA-binding activity was not detectable in untreated SW10 cells (Figure 2C).

### Oct-6 is induced by IFNβ and IFNγ in macrophages

**Figure 3:**
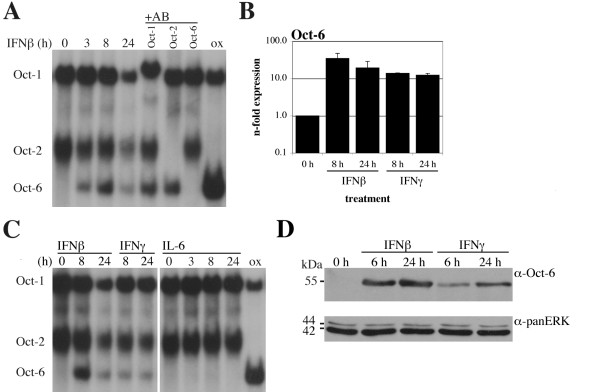
**Oct-6 is induced by IFNβ and IFNγ in macrophages**. WT BMMs were treated with IFNβ, IFNγ or IL-6 for the times indicated. (A, C) Whole cell extracts were analysed by EMSA as described in the legend of Figure 1 D. (A) DNA-binding complexes were identified by supershift of WT_8 h lysates with the respective antibodies (+AB), and by *Oct-6 *overexpression (ox) in a MEF cell line. (B) *Oct-6 *mRNA levels were determined by RT-qPCR as described in the legend of Figure 1A. Mean values ± SD of two experiments are shown. (D) Oct-6 was immunoprecipitated from whole cell extracts, panERK was used as an input control. (A, C, D) Representatives of at least two independent experiments are shown.

Macrophages are important cells of the immune system and their response to IFNβ is crucial in many infection and disease models. We thus tested bone marrow-derived macrophages (BMMs) for Oct-6 expression. Oct-6 mRNA and DNA-binding activity were clearly induced by IFNβ in macrophages (Figure 3A and 3B). Similar to MEFs, induction of Oct-6 DNA-binding was detectable with EMSA from 3 h IFNβ treatment onwards (Figure 3A), and Oct-6 was not detectable in untreated cells. Again, identity of Oct-6 was confirmed with anti-Oct-6 supershifts and, additionally, by demonstrating migration behaviour of overexpressed Oct-6 in EMSAs (Figure 3A). Consistent with what has been shown previously [30], constitutive Oct-1 and Oct-2 protein expression was observed in macrophages, as proven by supershift with specific antibodies (Figure 3A). Notably, Oct-6 protein expression and DNA-binding activity was considerably higher in macrophages than in fibroblasts and SW10 cells. Since IFNγ also induces several genes that are induced by IFNα/β, we tested whether Oct-6 is also induced by IFNγ. In addition, we analysed Oct-6 expression in response to interleukin-6 (IL-6), another cytokine that utilises the Jak/Stat signalling cascade but results in distinct cellular responses. IFNγ, which mainly activates Stat1, induced *Oct-6 *mRNA (Figure 3B), Oct-6 protein (Figure 3D) and DNA-binding activity (Figure 3C) to slightly lower levels as observed in response to IFNβ. In contrast, IL-6 which mainly signals *via *Stat3, did not result in detectable Oct-6 DNA-binding activity (Figure 3C).

### Oct-6 expression is dependent on Stat1 and partially dependent on Tyk2

**Figure 4:**
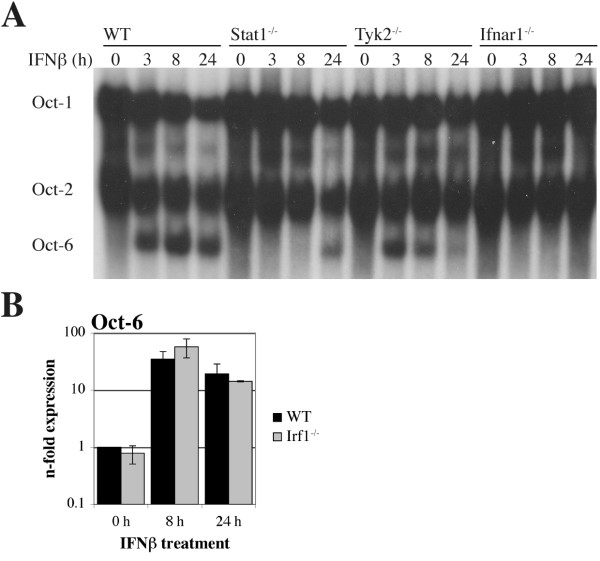
**Oct-6 induction by IFNβ depends on Stat1 and partially on Tyk2**. (A) WT, Stat1^-/- ^, Tyk2^-/- ^and Ifnar1^-/- ^BMMs were treated with IFNβ for the times indicated. Whole cell extracts were analysed by EMSA as described in the legend to Figure 1 D. A representative of at least two independent experiments per genotype is shown. (B) WT and Irf1^-/- ^BMMs were treated with IFNβ for the times indicated and *Oct-6 *mRNA expression was determined by RT-qPCR as described in the legend to Figure 1A. Mean values ± SD of two independent experiments are shown.

To determine if Oct-6 induction by IFN occurs *via *the canonical Jak/Stat signalling cascade, macrophages derived from mice deficient for specific Jak/Stat components were analysed. As expected, Oct-6 was not upregulated by IFNβ in cells lacking the IFNα/β receptor subunit Ifnar1, excluding the possibility that effects are mediated by any other component that might be present in the IFNβ preparation (Figure 4A). As in MEFs (Figure 1C), Oct-6 induction was partially dependent on the presence of Tyk2 (Figure 4A). Importantly, Oct-6 was hardly detectable in cells lacking Stat1 (Figure 4A), although low level of Oct-6 was consistently detectable after 24 h IFNβ treatment. Thus, Oct-6 induction occurs mainly in a Stat1-dependent manner. Stat1 induces transcription of interferon regulatory factor 1 (Irf1), a transcription factor that can bind to ISREs and is required for the regulation of a subset of IFN-responsive genes [31]. No difference in Oct-6 mRNA expression was found in Irf1-deficient as compared to WT cells, indicating direct involvement of Stat1 in the transcriptional activation of the Oct-6 gene (Figure 4B).

### Oct-6 is induced by poly(I:C) treatment and during viral infection via autocrine/paracrine IFNα/β signalling

**Figure 5:**
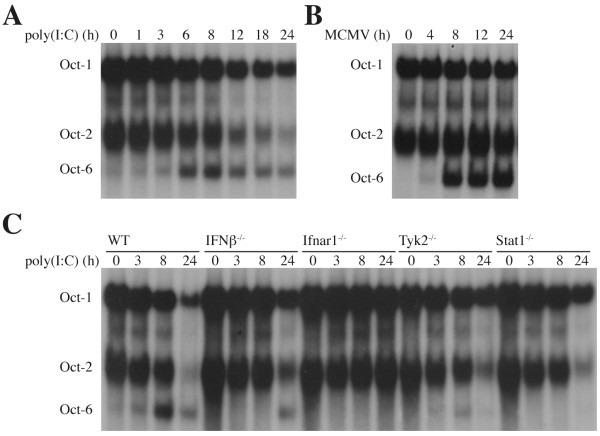
**Oct-6 is induced by poly(I:C) and during MCMV infections via autocrine/paracrine actions of IFNα/β**. (A, B) WT BMMs or (C) BMMs derived from mice of the indicated genotype were (A, C) treated with poly(I:C) or (B) infected with MCMV (MOI = 1) for the times indicated. Whole cell extracts were analysed by EMSA as described in the legend to Figure 1 D. Representatives of (A, B) three independent experiments and (C) at least two independent experiments per genotype are shown.

We next tested whether Oct-6 is also induced by pathogen recognition receptor (PRR) signalling in an IFNα/β-dependent or -independent manner [32,33]. To this end, macrophages were either treated with poly(I:C), a synthetic dsRNA analogue, or infected with Murine Cytomegalovirus (MCMV), both strong inducers of IFNα/β. As shown in Figure 5A and 5B, Oct-6 DNA-binding activity was upregulated in both scenarios. In order to determine whether Oct-6 is induced directly by PRR signalling, e.g. *via *NFκB or Irf3 transcription factor activation [32,33], we analysed Ifnar1-, IFNβ-, Tyk2- and Stat1-deficient macrophages in response to poly(I:C). Oct-6 expression was completely dependent on Ifnar1, demonstrating that Oct-6 expression after poly(I:C) treatment is mediated by autocrine/paracrine actions of IFNα/β (Figure 5C). Thus, in contrast to several other ISGs (e.g. IP-10), Oct-6 induction is strictly dependent on the presence of functional IFNα/β signalling. Unlike the residual Oct-6 induction observed after treatment with high amounts of exogenous IFNβ (Figure 4A), Oct-6 was not detectable in poly(I:C) treated Stat1^-/- ^cells (Figure 5C). Similarly, Tyk2 dependence was much more pronounced under these conditions than after treatment with high dose of exogenous IFNβ. Delayed induction was observed in cells deficient for IFNβ (Figure 5C), which is consistent with the prominent role of IFNβ in the IFNα/β amplification loop mainly described in the context of viral infections [34]. Similar results with respect to the requirement of the above analysed signalling molecules were obtained after MCMV infections (see additional file 2).

### Stat1 binds to the Oct-6 promoter

**Figure 6:**
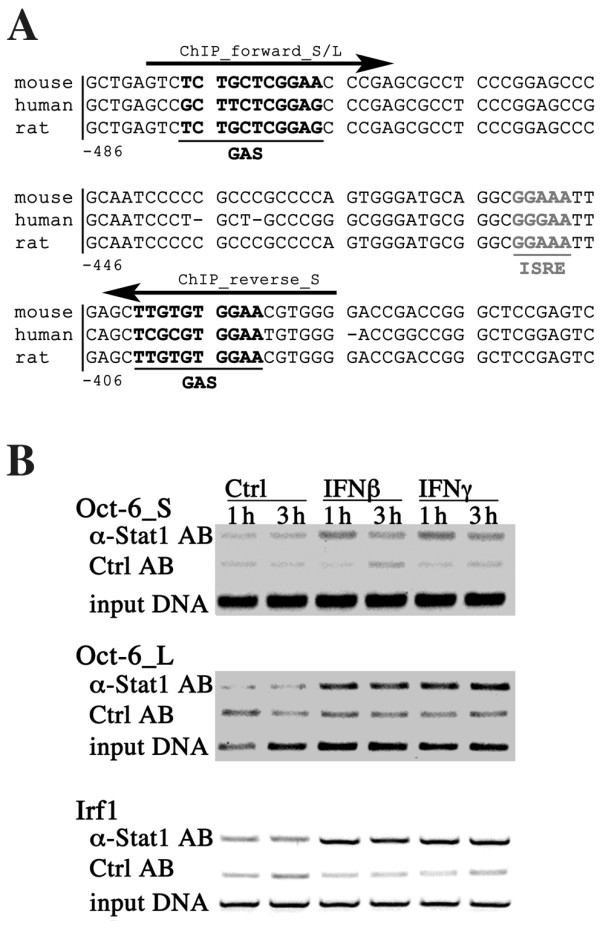
**Stat1 binds to a conserved region in the *Oct-6 *promoter containing predicted GAS and ISRE sites**. (A) Alignment of the selected conserved region upstream of the *Oct-6 *transcription start site (base counts correlate to the murine sequence). Potential GAS (black, bold) and ISRE (grey, bold) motifs are indicated. Position of primers used for the PCR reaction of the ChIP analysis are indicated by arrows. For the two PCRs, short (S) and long (L), the same forward primer was used, the reverse primer for the long PCR (not shown) is located 150 bp further downstream. (B) WT BMMs were treated with IFNβ (500 U/ml), IFNγ (200 U/ml) or were left untreated (Ctrl) for 1 h and 3 h. ChIP for Stat1 (α-Stat1 AB; nonspecific rabbit serum: Ctrl AB) was performed, followed by the two different PCRs for the *Oct-6 *promoter (Oct-6_S and Oct-6_L), and a PCR for the *Irf1 *promoter (Irf1) as a control. Representatives of two independent experiments are shown.

We next analysed the *Oct-6 *promoter for potential Stat1-binding sites (ISRE and GAS, respectively). Based on the assumption that conservation argues for functionality of consensus motifs, we analysed mouse, rat and human aligned sequences upstream of the respective transcription start sites. Within a homologous sequence part of *Oct-6*, one GAS (-477 to -468) and one ISRE (-412 to -409) was predicted by TFBS analysis using "Patch", a publicly available program scanning input sequences for potential TFBSs based on the Transfac database. Another GAS (-402 to -392) located in close proximity to the predicted ISRE was found by checking the sequences next to the predicted sites manually. The GAS elements (Figure 6A) show only one mismatch each compared to the consensus sequence (TTC(N)_2-4_GAA, [5]). The predicted ISRE (-412 to -409) seems rather imperfect when compared to the published consensus motif (GATTTC(N)_2_TTTCNY, [5]), but is identical to the Transfac annotated ISGF3 motif (GGAAA). To explore the possibility of Stat1-binding to this region, chromatin immunoprecipitation (ChIP) experiments were performed. In accordance with the mRNA induction, increased Stat1-binding to the *Oct-6 *promoter was observed in response to both IFNβ and IFNγ. Stat1-binding was observed with two different PCR reactions, encompassing the regions -481 to -387 or -481 to -243, respectively (Figure 6B, see Figure 6A for positions of primers used). As a control, Stat1-binding to a well-characterised binding region in the *Irf1 *promoter was performed and showed the expected pattern (Figure 6B) [35]. Thus, Stat1 directly binds to the *Oct-6 *promoter upstream of the transcription start site, most likely at the predicted GAS and/or ISRE site(s) in the region from position -481 to -387.

### IFNβ and poly(I:C) induced Oct-6 localises to the nucleus

**Figure 7:**
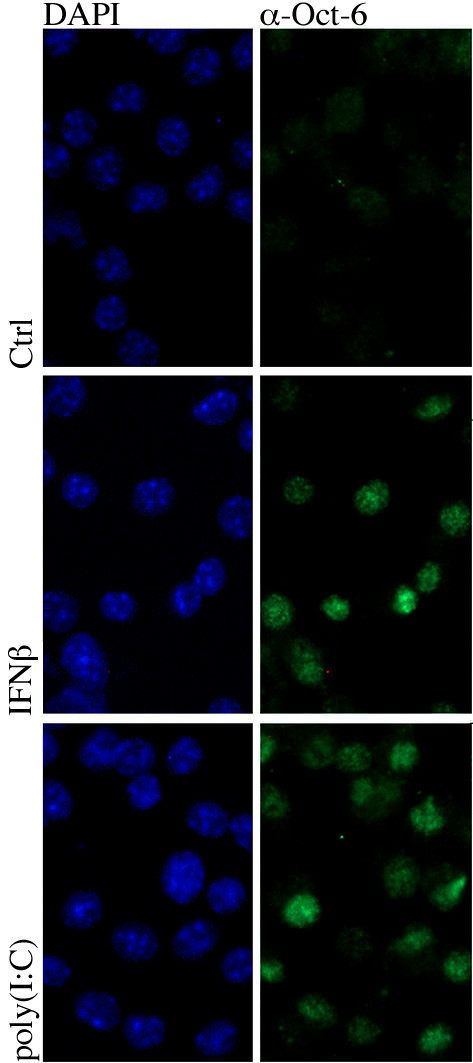
**Oct-6 localises to the nucleus in response to IFNβ or poly (I:C)**. WT BMMs were grown on glass slides, treated with IFNβ (middle panels) or poly(I:C) (lowest panels) for 6 h or left untreated (upper panels). Oct-6 was detected by indirect immunofluorescence (right panels), nuclei were stained with DAPI (left panels). Representatives of two independent experiments are shown.

Oct-6 contains a nuclear localisation and a nuclear export signal, both located in the POU homeodomain [36,37]. Although so far no post-translational modifications that direct Oct-6 to the nucleus have been identified, it has been hypothesised that its transcriptional activity can be controlled by regulating its subcellular localisation [36]. We therefore tested Oct-6 localisation after IFNβ and poly(I:C) treatment using immunofluorescence analysis. As expected, Oct-6 was not detected in untreated cells (Figure 7 top panel). In response to IFNβ and poly(I:C), Oct-6 staining was exclusively found in the nuclei (Figure 7). Hence, both IFNβ and poly(I:C) are sufficient to induce Oct-6 expression and nuclear localisation in macrophages.

### Absence of Oct-6 in marcrophages does not influence the expression of Egr2, Pmp22 and IFNαβ mRNAs

**Figure 8:**
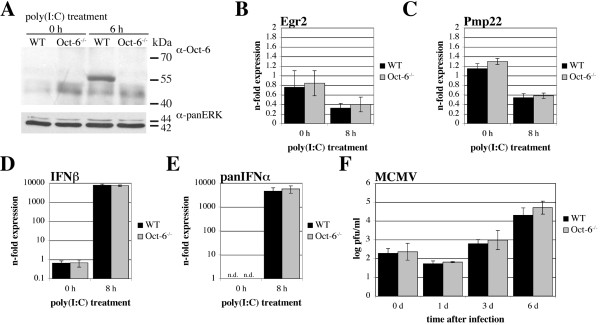
**Absence of Oct-6 in macrophages does not influence poly(I:C) induced *IFNβ *and *IFNα *mRNA expression and has no impact on MCMV replication**. (A - E) WT and Oct-6^-/- ^FLMs were treated with poly(I:C) or incubated with medium alone (0 h) for the times indicated. (A) Oct-6 was immunoprecipitated from whole cell extracts; panERK was used as an input control. (B - E) Expression levels were determined by RT-qPCR for (B) *Egr2*, (C) *Pmp22*, (D) *IFNβ*, and (E) *panIFNα *(all subtypes) using *Ube2d2 *as endogenous control. (B, C, D) Data are depicted relative to the WT 0 h control. (E) *panIFNα *mRNA could not be detected reliably in untreated cells (n.d.) and thus data normalised to the endogenous control only are depicted (not additionally calibrated to untreated cells). (B - E) Mean values ± SD of three independent experiments are shown. (F) WT and Oct-6^-/- ^FLMs were infected with MCMV (MOI = 1) for 90 min, washed with PBS and fresh medium was added. Supernatants were collected 0, 1, 3 and 6 days (d) after infection and virus titers were determined in a plaque forming assays using Stat1^-/- ^MEFs. Mean values ± SD of two independent experiments (each with FLMs from two embryos per genotype) are shown.

In order to investigate the biological function of Oct-6, we firstly analysed mRNA expression of potential target genes in the presence or absence of Oct-6. Since Oct-6^-/- ^mice die soon after birth, foetal livers were used to isolate Oct-6^-/- ^and WT macrophages, respectively. Similar to MEFs and BMMs, Oct-6 expression was not detectable by immunoprecipitation in untreated foetal liver-derived macrophages (FLMs), whereas it was clearly expressed after poly(I:C) treatment (Figure 8A). Inducibility of Oct-6 by poly(I:C) was similar to that observed in BMMs as analysed by EMSA (see additional file 3). Among the known target genes in Schwann cells, we analysed expression of *Egr2 *and peripheral myelin protein 22 (*Pmp22) *in response to poly(I:C). As shown in Figure 8B and 8C, poly(I:C) treatment resulted in a modest but Oct-6-independent down-regulation of both mRNAs. Octamer consensus motifs and Oct-1 have been implicated in negative regulation of *IFNα *and *IFNβ *gene expression [38]. Furthermore, transient transfection of *Oct-6 *resulted in a strong upregulation of type I IFN mRNAs (see additional file 4). However, we did not observe any difference in the poly(I:C)-induced expression of *IFNβ *or *IFNαs *(all subtypes, panIFNα) between Oct6^-/- ^and WT macrophages (Figure 8D and 8E). Similarly, no Oct-6-specific effects on the expression of type I IFNs were found in response DNA transfection in fibroblasts (see additional file 5).

Octamer proteins can influence viral replication [12] and the high induction of Oct-6 that we observed after MCMV infection in macrophages, prompted us to analyse MCMV replication in the presence or absence of Oct-6. WT and Oct-6-deficient macrophages were infected with MCMV and virus titer was measured over time. No difference in viral yield was observed after infection with a multiplicity of infection (MOI) of 1 (Figure 8F) or a MOI of 0.1 (see additional file 6).

### Oct-6 contributes to the regulation of a subset of genes in response to poly(I:C) in macrophages

In order to identify target genes of Oct-6 in the context of innate immune responses, WT and Oct-6^-/- ^FLMs were treated with poly(I:C) for 8 h and transcriptional responses were monitored using microarray analysis. A subset of genes (n = 200) displayed a significant, at least two-fold difference between WT and Oct-6^-/- ^macrophages after poly(I:C) treatment (*p *<*0.05*). About 60% of the genes were reduced, the rest enhanced in the absence of Oct-6, arguing for activating as well as repressing functions of Oct-6. Of the differentially expressed genes, 158 could be annotated, remaining probe sequences correlate to RIKEN cDNAs, or cDNAs of unknown function. About half of the differentially expressed genes (n = 96) could be grouped into classes/pathways using functional annotation and clustering (see additional file 7; see additional file 8 for the complete list of differentially expressed genes). The largest group was "Regulation of transcription" containing 15 genes, most of them down-regulated in the absence of Oct-6, such as *Ctnnd2*, *Rcor3 *and a number of zinc finger proteins. A related category "RNA splicing" contained 3 genes, *Sfrs14*, *Lsm10 *and *Sf3a1*, all of which showed reduced expression in the absence of Oct-6. Another category "Ubiquitin cycle" contained 6 genes, half of which were up-, half down-regulated. However, analyses for pathway enrichment did not yield any significant results, most likely because the number of differentially regulated genes was too low compared to the vast amount of GO-categories. Nevertheless, the data clearly show that Oct-6 is involved in the regulation of transcriptional responses to poly(I:C). As expected, in WT cells poly(I:C) treatment had a major effect on the transcriptome with around 3500 genes (out of 12220 genes included in the analysis) significantly regulated (*p *<*0.05*, minimal fold-change of 2). According to Gene Ontology (GO) annotation (GeneSpring Expression Analysis 7.3.1 tool, Agilent Technologies), genes involved in immune responses and cell death were significantly enriched in the set of genes up-regulated in response to poly(I:C) treatment (see additional file 9, sheet 1: Gene Ontology categories up-regulated by poly(I:C) treatment in WT cells). In the set of genes down-regulated in response to poly(I:C) treatment, genes involved in metabolism and cell cycle progression were significantly enriched (see additional file 9, sheet 2: GO categories down-regulated by poly(I:C) treatment in WT cells).

### Oct-6 regulates the expression of Stk40 and Lsm10 in response to poly(I:C)

**Figure 9:**
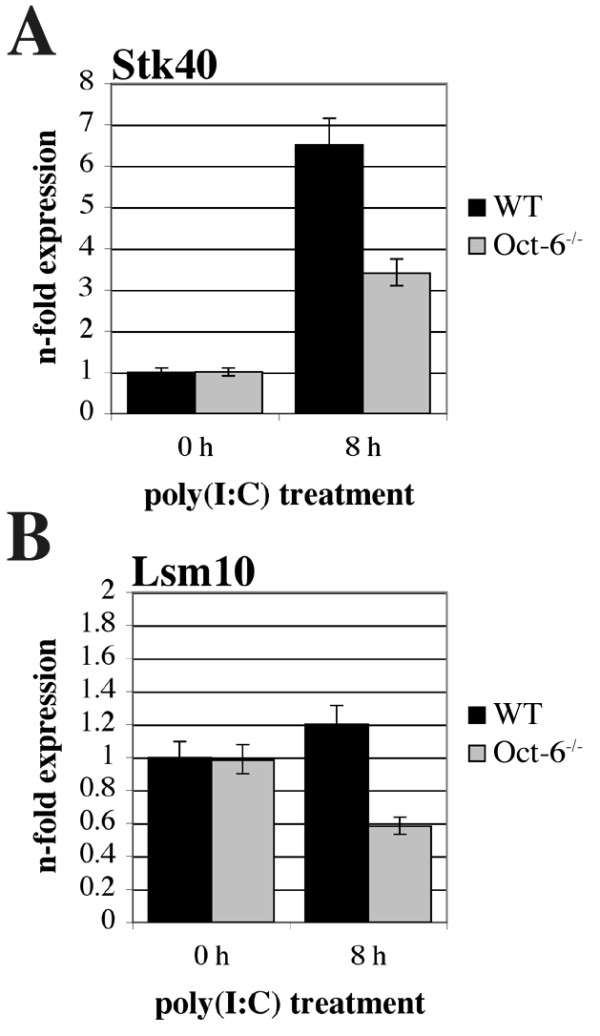
**Expression levels of *Stk40 *and *Lsm10 *after poly(I:C) treatment are reduced in the absence of Oct-6**. WT and Oct-6^-/- ^FLMs were treated with poly(I:C) for 8 h or incubated with medium alone (0 h). Expression levels of (A) *Stk40 *and (B) *Lsm10 *were determined by RT-qPCR using *Ube2d2 *as endogenous control. Data are depicted relative to untreated WT cells. Mean values ± SD of six experiments are shown.

In order to confirm the role of Oct-6 in the transcriptional regulation of poly(I:C) responses, we validated expression patterns of serine/threonine kinase 40 (*Stk40*) and U7 snRNP-specific Sm-like protein LSM10 (*Lsm10*) with RT-qPCR. Both genes showed around two-fold reduced expression levels in the absence of Oct-6 in the microarray experiment (*p *<*0.01*, see additional file 7). As shown in Figure 9A, *Stk40 *was induced about seven-fold in WT cells after poly(I:C) treatment, whereas the induction was only three-fold in Oct-6^-/- ^macrophages. *Lsm10 *was not significantly influenced by poly(I:C) treatment in WT cells, but its expression was reduced by two-fold in the absence of Oct-6 (Figure 9B). For both genes, differences in expression between WT and Oct-6^-/- ^macrophages after poly(I:C) treatment were again highly significant (*p *<*0.01*).

## Discussion

In this study we report for the first time expression of Oct-6 in fibroblasts and macrophages. We show that Oct-6 is induced by IFNβ and IFNγ, but not by IL-6. Expression of Oct-6 in response to IFNβ occurs mainly *via *the canonical Jak/Stat signalling cascade and is dependent on the presence of Stat1 and to a lesser extent on Tyk2. Notably, we observed delayed and low levels of Oct-6 induction in the absence of Stat1 in response to high dose of exogenous IFNβ, suggesting that additional IFN activated factors can mediate Oct-6 induction. Oct-6 is also expressed during viral infection and after treatment with the synthetic dsRNA analogue poly(I:C), in both cases mediated by autocrine/paracrine IFNα/β signalling. Using ChIP technology, we show that Stat1 directly binds to the Oct-6 promoter at around 387 to 481 bp upstream of the transcription start site, a region containing three conserved Stat1 consensus binding sites (i.e. two GAS sites and one imperfect ISRE). The presence of GAS and ISRE in the *Oct-6 *promoter is consistent with the responsiveness to IFNγ and IFNβ, a feature that is shared with several other IFN responsive genes. In contrast to the upstream Stat1-binding region defined herein, an enhancer element around 12 kb downstream of the *Oct-6 *gene mediates *Oct-6 *expresssion during Schwann cell development. Deletion of this enhancer element completely abolishes *Oct-6 *expression in Schwann cells without affecting its expression in other cell types [25,39]. Schwann cell-axonal contact induces *Oct-6 *expression by possibly multiple pathways [40], but transcription factors and co-activators involved are still poorly characterised. NFκB is required but not sufficient for Oct-6 expression [41] and it is at present unknown how the different signals input on the Oct-6 enhancer element. Thus, the mechanism of gene induction is clearly different for IFN- and Schwann cell-axonal contact-induced Oct-6 expression and involves distinct regulatory regions. Potential cross-influence and regulation in other cell types remain to be investigated.

Except for the ubiquitous Oct-1, expression of all POU domain proteins is regulated in a developmental and/or cell differentiation-dependent manner, with some members being lineage-specifying factors [12,42,43]. Inducibility of Oct-6 in the context of innate immune responses adds a new facet to POU domain protein biology and raises the question if Oct-6 is unique in this regard or if other members display similar as yet unrecognised modes of regulation.

Using WT and Oct6-deficient foetal liver-derived macrophages, we demonstrate that IFNβ and poly(I:C) treatment is sufficient to induce Oct-6 expression and nuclear localisation. We could demonstrate a functional role of Oct-6 in poly(I:C) induced transcriptional responses using microarray analysis of WT compared to Oct-6-deficient cells. 200 genes were at least two-fold differentially expressed between WT and Oct-6-deficient cells after poly(I:C) treatment. Genes affected by the absence of Oct-6 were diverse with respect to functional annotation and no specific pathway appeared as Oct-6 regulated. Using RT-qPCR we confirmed Oct-6-dependent gene regulation for *Lsm10 *and *Stk40*. Interestingly, the effect of Oct-6 on gene expression was different for these two genes. In the case of *Stk40*, Oct-6 was needed for efficient upregulation, whereas it was required for the maintenance of *Lsm10 *expression after poly(I:C) treatment. The function of Stk40, also known as SINK homologous serine/threonine kinase (SHIK) and Lyk4, is poorly characterised. Very recently, *Stk40 *has been identified as Oct-4 target gene that is required for extraembryonic endoderm differentiation [44]. On the other hand, overexpressed *Stk40 *was shown to inhibits TNF-induced NFκB- and p53-mediated transcription [45]. Lsm10 is a component of the U7 small nuclear ribonucleoprotein (snRNP) complex, which is involved in the formation of the 3' end processing of canonical histone mRNAs [46,47]. Against this background, it can be speculated that Oct-6 influences chromatin remodelling and cell cycle progression. More detailed gene expression analyses, in particular considering kinetic aspects, will certainly be required in order to fully characterise the role of Oct-6 in innate immune responses. It will also be of major interest to determine the role of Oct-6 in innate immunity *in vivo*. However, *s*ince Oct-6 deficient animals die soon after birth [21,22] these studies will have to await the availability of conditional knockout mice.

Interestingly, absence of Oct-6 in macrophages did not affect the expression of *Egr2 *and *Pmp22*, genes that are regulated by Oct-6 in Schwann cells [23,48]. Thus, target genes of Oct-6 differ between cell types and/or stimuli. Dependent on specific DNA elements and the presence of distinct co-activators, POU proteins can assume different conformations [49]. With respect to co-activators, the SRY box protein Sox10 cooperates with Oct-6 in Schwann cells and glial cells [23,50,51]. It is reasonable to assume that distinct transcriptional co-activators are present or activated in macrophages, fibroblasts and Schwann cells and that these account for the differences in target gene expression.

It seems worth considering our results also in the context of neuropathologies. We demonstrate that Oct-6 is induced by IFNβ in the murine Schwann cell line SW10. Oct-6 protein expression was very low in this cell line, but the potency of IFNs to induce Oct-6 might be interesting enough to prompt studies on primary Schwann cells and *in vivo*. In particular in the context of inflammatory and infection-induced neuropathologies, where IFNs are produced and Schwann cells de-differentiate and develop again to a myelinating phenotype. We hypothesise that IFN application or stimulation by e.g. viral infection can influence peripheral and possibly central nervous system myelination by triggering Oct-6 expression.

## Conclusions

We identify *Oct-6 *as an ISG and inducible protein in cell types where it has not been reported yet. In addition to the known Oct-6 function mainly in developmental processes, our report places Oct-6 as a transcriptional (co-) activator in the innate immune response repertoire. Furthermore, our results provide new insights into *Oct-6 *gene regulation with a potential impact on the control of nerve myelination.

## Methods

### Mice and cells

Mice deficient for Ifnar1 [52], Tyk2 [53], Stat1 [54], Irf1 [55] and IFNβ [56] were crossed for at least ten generations onto C57BL/6 background. Wild type (WT) mice (C57BL/6) were purchased from Charles River Laboratories. Mice were housed under specific pathogen-free conditions according to FELASA guidelines, except for Oct-6^+/- ^mice which were housed conventionally. Oct-6^+/- ^mice were of mixed background [21], Oct-6^+/+ ^littermates were used as WT controls. All animal experiments were discussed and approved by the institutional ethics committee and the Austrian laws (GZ 68.205/0204-C/GT/2007 and GZ 68.205/0233-II/10b/2009). Bone marrow-derived macrophages (BMMs) were grown in the presence of L929 conditioned medium as described [57] and used for experiments on day 7 after isolation. Foetal liver-derived macrophages (FLMs) were isolated/grown by culturing foetal liver cell suspensions (day 13.5-14.5 post conception) under the same conditions as BMMs and were used for experiments on day 6 after isolation. Primary murine embryonic fibroblasts (pMEFs) were isolated and grown from individual embryos at day 13.5-14.5 post conceptionem according to standard procedures [58]. MEF cell lines were grown as described [59]. SW10 cells were from ATCC (Cat. No. CRL-2766; [29]) and propagated as recommended.

### Reagents, treatments and infections

Cells were treated for the indicated time points with IFNβ, IFNγ or IL-6 (all purchased from Calbiochem), or with polyinosinic-polycytidylic acid (poly(I:C), GE Healthcare). IL-6 was used at a concentration of 125 ng/ml, poly(I:C) at 50 μg/ml, IFNβ and IFNγ at 1000 U/ml (if not stated otherwise). Infections with Murine Cytomegalovirus (MCMV) and plaque assays were done as described previously [59].

### Whole cell extracts and immunoprecipitations (IP)

Cells were lysed in 50 mM Tris.HCl pH8, 150 mM NaCl, 0.5% Nonidet P-40, 10% glycerol, 2 mM DTT, 0.1 mM EDTA, 0.2 mM Na_3_VO_4_, 25 mM NaF, 1 μg/ml aprotinin, 1 μg/ml leupeptin and 1 mM PMSF. Cell debris was removed by centrifugation. 1 mg whole cell extract/ml was incubated overnight with 2 μg anti-Oct-6 antibody (C-20, Santa Cruz) and purified with protein-G PLUS agarose (Santa Cruz). Precipitates were separated on 8% SDS polyacrylamide gels. Proteins were blotted onto nitrocellulose membranes (GE Healthcare), detection was performed with a previously described rabbit polyclonal anti-Oct-6 antibody [60]. As an input control for IPs, whole cell extracts were analysed for panERK (pan-extracellular signal regulated kinases) expression. panERK antibody was from BD Transduction Laboratories. Anti-rabbit- and anti-mouse-IgG horse-raddish peroxidase-conjugated secondary antibodies and the ECL™-detection system were from GE Healthcare.

### Electrophoretic mobility shift assays (EMSAs)

EMSAs were done as described previously [61] using 15 μg whole cell extract and an octamer consensus motif containing oligonucleotide [62]. For supershifts, cell extracts were incubated with 1 μl of the respective antibody prior to the binding reaction: anti-Oct-6 (C-20), anti-Oct-1 (12F11), and anti-Oct-2 (C-20) (all Santa Cruz).

### Immunofluorescence

Cells were grown and stimulated on glass slides, and fixed with 4% formaldehyde for 15 min (Histofix, Roth). Formaldehyde was quenched by glycine (100 mM, 15 min), cells were permeabilised by methanol treatment (-20°C, 5 min). Nonspecific binding was blocked with 1% BSA in PBS for 1 hour. Oct-6 was detected by incubating the slides with anti-Oct-6 antibody (C-20, Santa Cruz; 4 μg/ml in blocking solution; 4°C overnight) and a fluorescently labelled a-goat IgG secondary antibody (Alexa-Fluor™ 488; 1:200 in PBS; 1 hour at room temperature). Goat IgG (Invitrogen) was used as isotype control. Nuclei were counterstained with DAPI (100 ng/ml).

### Alignment of Oct-6 upstream sequences and transcription factor binding site (TFBS) prediction

For the alignments, mouse, rat and human sequences from +1 to -5 kb were used (Blast 2 sequences: http://blast.ncbi.nlm.nih.gov/bl2seq/wblast2.cgi; March 2008). Mouse sequence accession number NC_000070.5 (M.m. C57BL/6, chromosome 4, reference assembly; base 124334896 = +1 on the +strand). Human sequence accession number NC_000001_10 (H.s. chromosome 1, GRCh37 primary reference assembly; base 38512450 = +1 on the -strand). Rat sequence accession number NC_005104.2 (R.n., chromosome 5, reference assembly; base 143981547 = +1 on the +strand). Homologous sequence parts, i.e. -4367 to -4500, -1598 to -3233, and -161 to -637 (base counts correlate to the murine sequence), were submitted to TFBS analysis analysed using "Patch" (http://www.gene-regulation.com/pub/programs.html; August 2008), which scans input sequences for potential TFBSs based on the Transfac database. A number of Stat1- and ISGF3-binding sites were predicted for each sequence part. We decided to concentrate on the homologous sequence part nearest to the transcription start site (-161 to -637) of *Oct-6*, based on a report showing that the regions -500 bp upstream of the transcription start site of IFN-inducible genes are enriched in predicted binding sites for Stat1 and ISGF3 [63].

### Chromatin immunoprecipitation (ChIP)

ChIP for Stat1 was performed as described [35] with minor modifications. Sonication (Sonopuls HD70, MS72 sonotrode; Bandelin) was performed at 50% power and 90% duty cycle for 10 times 15 sec with 1 min break between the pulses. Equal amounts of lysate were used for Stat1 IP (4 μl Stat1-C antibody/500 μl lysate; a kind gift from Pavel Kovarik, MFPL, University of Vienna; [64]) and a control reaction using nonspecific rabbit serum (Sigma). DNA was isolated following a phenol:chloroform extraction protocol and subjected to PCR analysis. PCRs were run in a final volume of 25 μl containing 300 nM primer, 2 mM MgCl_2_, 200 μM dNTPs (Fermentas), 1× Biotaq buffer, 2 U Biotaq DNA polymerase (Agrobiogen) under following cycling conditions: 5 min at 95°C for initial denaturation, followed by 35 cycles of 95°C for 30 sec and 61°C for 1 min. PCRs were done from all samples of the anti-Stat1 IP, the control IP (nonspecific rabbit serum) and from an aliquot of the initial sample input prior to the IP (input DNA). Following primers were used: ChIP_Oct6-F: GTCTCTGCTCGGAACCCGA, ChIP_Oct6_S-R: CCCACGTTCCACACAAGCT, ChIP_Oct6_L-R: GCCCGCGTACACATTCAC; ChIP_Irf1-F: GCACAGCTGCCTTGTACTTCC, ChIP_Irf1-R: TCGGCCTCATCATTTCGG.

### RNA isolation and reverse transcription (RT)

Total RNA was isolated following the TRIZOL (Invitrogen) protocol. Prior to cDNA synthesis, RNA was treated with 1 U/μg RNA RQ1 DNase I (Promega) in order to digest contaminating genomic DNA. cDNA was prepared from 1 μg total RNA per 20 μl reaction using the iScript First Strand cDNA synthesis kit (BioRad), including controls for DNA contamination (reactions without addition of reverse transcriptase).

### Real-time quantitative PCR (qPCR) analysis of gene expression

Target gene expression was assessed by qPCR with ubiquitin-conjugating enzyme E2D2 (*Ube2d2*) as endogenous control gene. Assays for *IFNβ *and *Ube2d2 *were described previously [59]. Taqman^® ^probes labelled with 6-carboxyfluorescein (FAM) at the 5'end, and a black-hole-quencher (BHQ1) at the 3'end were used. EvaGreen (Biotium) assays were used for the quantification of *Oct-6*, *Egr2*, *Pmp22*, *Lsm10*, and *Stk40*. Following primers were used (5' to 3'): panIFNa:fwd-CCACAGGATCACTGTGT(A/T)CCTGAGA, rev-CTGATCACCTCCCAGGCACAG, probe-AG+AA+GAA+A+C+AC+AG+CC (locked nuclear acids (LNAs) are indicated by a "+" in front of the respective base; [65]);

*Oct-6*: fwd-AGGTCCTGTTGGAGATGATATGTT, rev-TTGGGAAATGAATTGTCAAGAAA;

*Egr2*: fwd-GGTGACCATCTTCCCCAATG, rev-TTGATCATGCCATCTCCCG;

*Pmp22*: fwd-CCGGTTTTACATCACTGGATTCT, rev-TGTAGATGGCCGCTGCACT;

*Lsm10*: fwd-CCTCCAAAAGGCCATGAGACT, rev-CGGGAGTTGGCTCAGAACAC;

*Stk40*: fwd-CTCTCAGTGCCATCATTGCATC, rev-CACCTTTGCCTCCTGGGA.

Taqman qPCR assays were run in a final volume of 25 μl containing 300 nM primer (Invitrogen), 100 nM probe (Sigma or Metabion), 200 μM dNTPs (Fermentas), 4 mM MgCl_2_, 1× HotFire buffer B, and 1U HotFire DNA polymerase (all Solis BioDyne). The conditions were the same for the EvaGreen assays, except for the use of 0.2× EvaGreen dye instead of the probe in the presence of only 2.5 mM MgCl_2_. All qPCRs were run on a Mastercycler^® ^ep Realplex (Eppendorf) applying following cycling conditions: 15 min at 95°C for initial denaturation, then 40 to 45 cycles of 95°C for 20 sec and 60°C for 1 min. For EvaGreen assays, the PCR was followed by a melting curve analysis in order to confirm assay specificity. Data were analysed using the Realplex software (Eppendorf) and relative target gene expression levels (i.e. n-fold expression levels) were calculated following the standard curve method [66,67].

Statistical analysis of RT-qPCR data. RT-qPCR gene expression data were investigated for differences among genotypes and time after challenge. Univariate regression was calculated with the log of the transformed target to endogenous control gene expression ratio as dependent variable. Linear contrasts were encoded such that for each time point Oct6^-/- ^were compared to WT cells. Differences among experiments were controlled for. Data were analysed with SPSS 17.0 for Mac OS-X.

### Microarray analysis

WT and Oct-6^-/- ^FLMs were treated with 50 μg/ml poly(I:C) for 8 hours in three independent experiments. RNA integrity was assessed by capillary electrophoresis using a Bioanalyser2100 (Agilent Technologies), and photometric analysis (OD260 nm/280 nm ration of ~2.1 for all samples). RNA integrity numbers (RIN) ranged between 9.2 and 9.4 indicating high quality of RNA samples. ABI1700 Mouse Genome Survey Microarrays (Applied Biosystems) in combination with the RT direct labelling kit (Applied Biosystems) were used according to the manufacturer's recommendations to generate gene expression profiles. 20 μg of input total RNA was used for direct labelling and microarray hybridisation. Data transformation and normalisation: expression values less than 10 were set to 10. Data were normalised to the 50^th ^percentile (intra-array normalisation) and each gene was normalised to the median expression (inter-array normalisation). Data were pre-filtered based on the signal to noise ratio (cut-off level: signal above noise > two-fold in all samples of the respective biological replicate group) and non-changing genes (normalised expression levels from 0,667 to 1,334 in at least 4 of 4 conditions) were subtracted. The remaining data set was tested for differentially expressed genes using ANOVA (GeneSpring Expression Analysis 7.3.1 tool, Agilent Technologies). A p-value of *p < 0.05 *(Welch t-test) was considered significant. Expression differences of at least two-fold were considered relevant. Not fully annotated probes, i.e. probes that did not correspond to a NCBI RefSeq, EST or RIKEN cDNA, were excluded. Functional annotation and clustering of the differentially regulated genes was performed using the Gene Functional Classification tool of the database for annotation, visualisation and integrated discovery (DAVID; http://david.abcc.ncifcrf.gov/[68,69]). The array data have been deposited in the Gene Expression Omnibus database, http://www.ncbi.nlm.nih.gov/geo (GEO accession no. GSE22691).

## List of abbreviations

BMMs: bone marrow-derived macrophages; FLMs: foetal liver-derived macrophages; Egr2: early growth response protein 2; GAS: IFNγ activated sequence; IFN: interferon; Ifnar1: IFNα/β receptor 1; ISRE: interferon stimulated response element; Lsm10: U7 snRNP-specific Sm-like protein LSM10; MCMV: Murine Cytomegalovirus; pMEFs: primary murine embryonic fibroblasts; Pmp22: peripheral myelin protein 22; POU-family: Pit-Oct-Unc-family; Stat1: signal transducer and activator of transcription 1; Stk40: serine/threonine kinase 40; Tyk2: tyrosine kinase 2; WT: wildtype.

## Authors' contributions

EH performed most of the experiments, participated in the design of the study and wrote the first draft of the manuscript. UR performed the mouse routine and experimental breeding, contributed to the establishment of FLM cultures and helped with the microscopy. CGa initially identified *Oct-6 *as an IFN regulated gene and performed some of the experiments with fibroblasts. CGu supervised the microarray experiment and performed the statistical analysis. DM provided the Oct-6^+/- ^mice and the Oct-6 antibodies and participated in the interpretation of initial data. MM contributed to the design and co-ordination of the study. BS co-ordinated and designed the study and wrote the manuscript together with EH and MM. All authors read and approved the final manuscript.

## Supplementary Material

Additional file 1 **Oct-6 protein is expressed in pMEFs in response to IFNβ treatment**. Bandshift assays including supershifts with α-Oct-1, α-Oct-2 and α-Oct-6 antibodies and Oct-6^-/- ^MEFs as controls. Click here for file

Additional file 2 **Expression of Oct-6 during MCMV infection is largely dependent on type I IFN and Jak/Stat signalling**. Bandshift assays of whole cell extracts from WT, IFNβ^-/-^, Ifnar1^-/-^, Tyk2^-/-^, and Stat1^-/- ^macrophages infected with MCMV. Click here for file

Additional file 3 **Oct-6 DNA-binding activity in response to IFNβ or poly(I:C) treatment in foetal liver- and bone marrow-derived macrophages**. Click here for file

Additional file 4 **Overexpression of Oct-6 in pMEFs enhances the expression of *IFNβ *and *IFNα *mRNAs, but does not influence the expression of *Egr2 *and *Pmp22 *mRNAs**. Click here for file

Additional file 5 **Absence of Oct-6 does not influence the expression patterns of *panIFNα*, *IFNβ*, *Egr2 *and *Pmp22 *mRNAs upon DNA transfection in MEFs**. Comparison of transfected WT and Oct6-deficient foetal liver-derived macrophages. Click here for file

Additional file 6 **Absence of Oct-6 in macrophages has no impact on MCMV replication at a MOI of 0.1**. Comparison of MCMV replication in WT and Oct-6-deficient foetal liver-derived macrophages at a lower MOI. Click here for file

Additional file 7 **Differentially expressed genes as determined by microarray analysis that could be functionally annotated (96 out of 200; WT *vs*. Oct-6^-/-^, at least 2-fold difference, *p *<*0.05*)**. Click here for file

Additional file 8 **Complete list of differentially expressed genes (WT *vs*. Oct-6^-/-^, at least 2-fold difference, *p *<*0.05*)**. Click here for file

Additional file 9 3A (sheet 1): Gene Ontology categories up-regulated by poly(I:C) treatment in WT cells (*p *<*0.05*); 3B (sheet 2): Gene Ontology categories down-regulated by poly(I:C) treatment in WT cells (*p *<*0.05*). Click here for file
