# Supplementary material for: Octamer-binding factor 6 (Oct-6/Pou3f1) is induced by interferon and contributes to dsRNA-mediated transcriptional responses
Source: BMC Cell Biol. 2010 Aug 5;11:61. doi: 10.1186/1471-2121-11-61 (PMC2924845; doi:10.1186/1471-2121-11-61)
Supplement: Additional file 1 — Oct-6 protein is expressed in pMEFs in response to IFNβ treatment. Bandshift assays including supershifts with α-Oct-1, α-Oct-2 and α-Oct-6 antibodies and Oct-6-/- MEFs as controls. [file 1471-2121-11-61-S1.PDF]

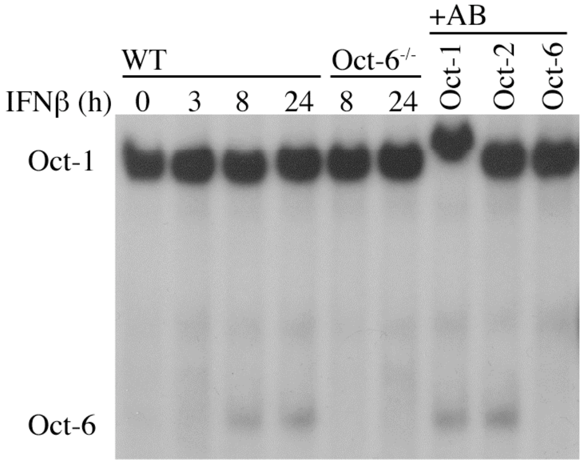

Additional file 1.

Oct-6 DNA-binding activity is induced in pMEFs in response to IFNβ treatment. WT and Oct-6$^{-/-}$ MEFs were treated with IFNβ (1000 U/ml) for the times indicated. Whole cell extracts were analysed by bandshift assays using an octamer motif-containing oligonucleotide; supershifts for Oct-1, Oct-2 and Oct-6 were performed with the respective antibodies (+AB).
