# Supplementary material for: Octamer-binding factor 6 (Oct-6/Pou3f1) is induced by interferon and contributes to dsRNA-mediated transcriptional responses
Source: BMC Cell Biol. 2010 Aug 5;11:61. doi: 10.1186/1471-2121-11-61 (PMC2924845; doi:10.1186/1471-2121-11-61)
Supplement: Additional file 2 — Expression of Oct-6 during MCMV infection is largely dependent on type I IFN and Jak/Stat signalling. Bandshift assays of whole cell extracts from WT, IFNβ-/-, Ifnar1-/-, Tyk2-/-, and Stat1-/- macrophages infected with MCMV. [file 1471-2121-11-61-S2.PDF]

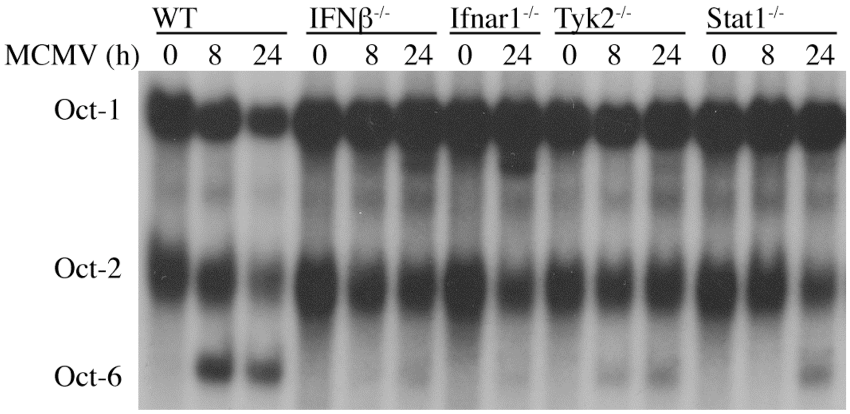

Additional file 2.
Expression of Oct-6 during MCMV infection is largely dependent on type I IFN and Jak/Stat signalling. WT, IFNβ$^{-/-}$, Ifnar1$^{-/-}$, Stat1$^{-/-}$, or Tyk2$^{-/-}$ BMMs were infected with MCMV (MOI=1) for the times indicated. Whole cell extracts were analysed by bandshift assays using an octamer motif-containing oligonucleotide.
