# Supplementary material for: Octamer-binding factor 6 (Oct-6/Pou3f1) is induced by interferon and contributes to dsRNA-mediated transcriptional responses
Source: BMC Cell Biol. 2010 Aug 5;11:61. doi: 10.1186/1471-2121-11-61 (PMC2924845; doi:10.1186/1471-2121-11-61)
Supplement: Additional file 3 — Oct-6 DNA-binding activity in response to IFNβ or poly(I:C) treatment in foetal liver- and bone marrow-derived macrophages. [file 1471-2121-11-61-S3.PDF]

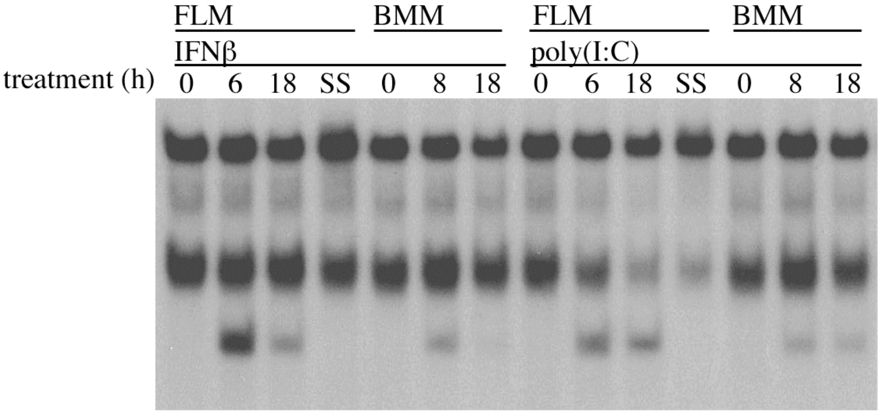

Additional file 3.

Oct-6 DNA-binding activity in response to IFNβ or poly(I:C) treatment is also induced in foetal liver-derived macrophages (FLMs). FLMs and BMMs were treated with IFNβ (1000 U/ml) or poly(I:C) (50 μg/ml) for the times indicated. Whole cell lysates were analysed by a bandshift assay using an octamer motif-containing oligonucleotide (SS: supershift of the FLM_IFNβ_6h and FLM_poly(I:C)_6h lysate with an α-Oct-6 antibody).
