# Supplementary material for: Octamer-binding factor 6 (Oct-6/Pou3f1) is induced by interferon and contributes to dsRNA-mediated transcriptional responses
Source: BMC Cell Biol. 2010 Aug 5;11:61. doi: 10.1186/1471-2121-11-61 (PMC2924845; doi:10.1186/1471-2121-11-61)
Supplement: Additional file 4 — Overexpression of Oct-6 in pMEFs enhances the expression of IFNβ and IFNα mRNAs, but does not influence the expression of Egr2 and Pmp22 mRNAs. [file 1471-2121-11-61-S4.PDF]

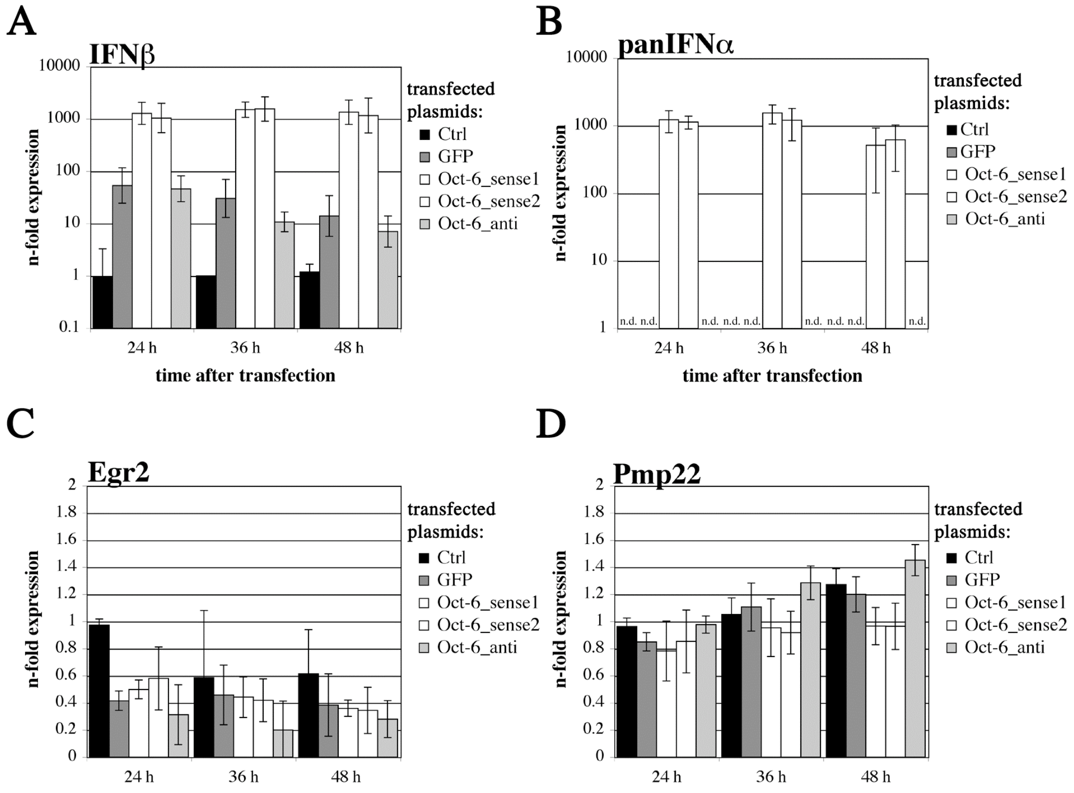

Additional file 4.

Overexpression of Oct-6 in pMEFs enhances the expression of IFNβ and IFNα mRNAs, but does not influence the expression of Egr2 and Pmp22 mRNAs. MEFs were transfected with the following plasmids (10 μg each): enhanced-GFP expression vector (GFP), two different Oct-6 expression plasmids (Oct-6_sense1/2), and a plasmid containing the Oct-6 cDNA in antisense direction (Oct-6_anti). As a control untransfected cells (Ctrl) were analysed. Expression levels of (A) IFNβ, (B) panIFNα (all IFNα subtypes), (C) Egr2 and (D) Pmp22 mRNAs were determined 24 h, 36 h, and 48 h after transfection by RT-qPCR using Ube2d2 as endogenous control. (A, C, D) Data are depicted relative to the Ctrl at 24 h. (B) panIFNα mRNA could not be detected reliably in untreated cells (n.d.) and thus data normalised to the endogenous control are depicted (not additionally calibrated to untreated cells). Mean values ± SD of three independent experiments are shown.

Plasmids

The CMV enhancer-driven Oct-6 expression plasmid was described previously (based on pEVRF0 plasmid (1), Oct-6_sense1). Sense and antisense sequence of the Oct-6 cDNA were cloned into the EF1α-promoter driven expression vector pEFZeo (a kind gift from Pavel Kovarik, MFPL, University of Vienna (2); Oct-6_sense2 and Oct-6_anti). The Oct-6 coding sequence was PCR-amplified from genomic DNA with primers containing restriction sites for BamHI at the 5'ends of the forward (Oct6_BamHI-F: TACGGATCCCGCAGACGGAGCGAGGCG) and reverse (Oct6_BamHI-R: TAGGGATCCGAACCCAGTCCGCAGGGTCAC) primer under the following conditions: in 50 μl final volume 300 nM primer (Invitrogen), 5% DMSO (Sigma), 200 μM dNTPs (Fermentas), 5 U Pfu DNA polymerase, 1x Pfu buffer incl. $MgSO_4$ (2 mM final concentration; all MBI Fermentas) were used, running following PCR program: 95°C for 3 min, 35 cycles of 95°C for 30 sec, 67°C for 30 sec, 72°C for 2 min, and a final extension step of 72°C

for 7 min. The 1.4 kb insert was blunt-ligated into EcoRV digested pEFZeo. Sense and antisense insertion was checked by XhoI digestion. Correct amplification and cloning was confirmed by sequencing analysis (reference sequence accession number NM_011141.1). The enhanced GFP expression plasmid (GFP) was supplied with the transfection kit (Amaxa/ Lonza) as a positive control. Plasmids were purified using the Jetstar Plasmid Midiprep 2.0 Kit (Genomed). Plasmids were transfected into primary fibroblasts using the manufacturer's protocols (MEF1 nucleofector kit, Amaxa/Lonza)

References

(1) **Meijer, D., A. Graus, and G. Grosveld.** 1992. Mapping the transactivation domain of the Oct-6 POU transcription factor. Nucleic Acids Res **20:**2241-7.

2) **Kovarik, P., M. Mangold, K. Ramsauer, H. Heidari, R. Steinborn, A. Zotter, D. E. Levy, M. Muller, and T. Decker.** 2001. Specificity of signaling by STAT1 depends on SH2 and C-terminal domains that regulate Ser727 phosphorylation, differentially affecting specific target gene expression. EMBO J **20:**91-100.
