# Supplementary material for: Octamer-binding factor 6 (Oct-6/Pou3f1) is induced by interferon and contributes to dsRNA-mediated transcriptional responses
Source: BMC Cell Biol. 2010 Aug 5;11:61. doi: 10.1186/1471-2121-11-61 (PMC2924845; doi:10.1186/1471-2121-11-61)
Supplement: Additional file 5 — Absence of Oct-6 does not influence the expression patterns of panIFNα, IFNβ, Egr2 and Pmp22 mRNAs upon DNA transfection in MEFs. Comparison of transfected WT and Oct6-deficient foetal liver-derived macrophages. [file 1471-2121-11-61-S5.PDF]

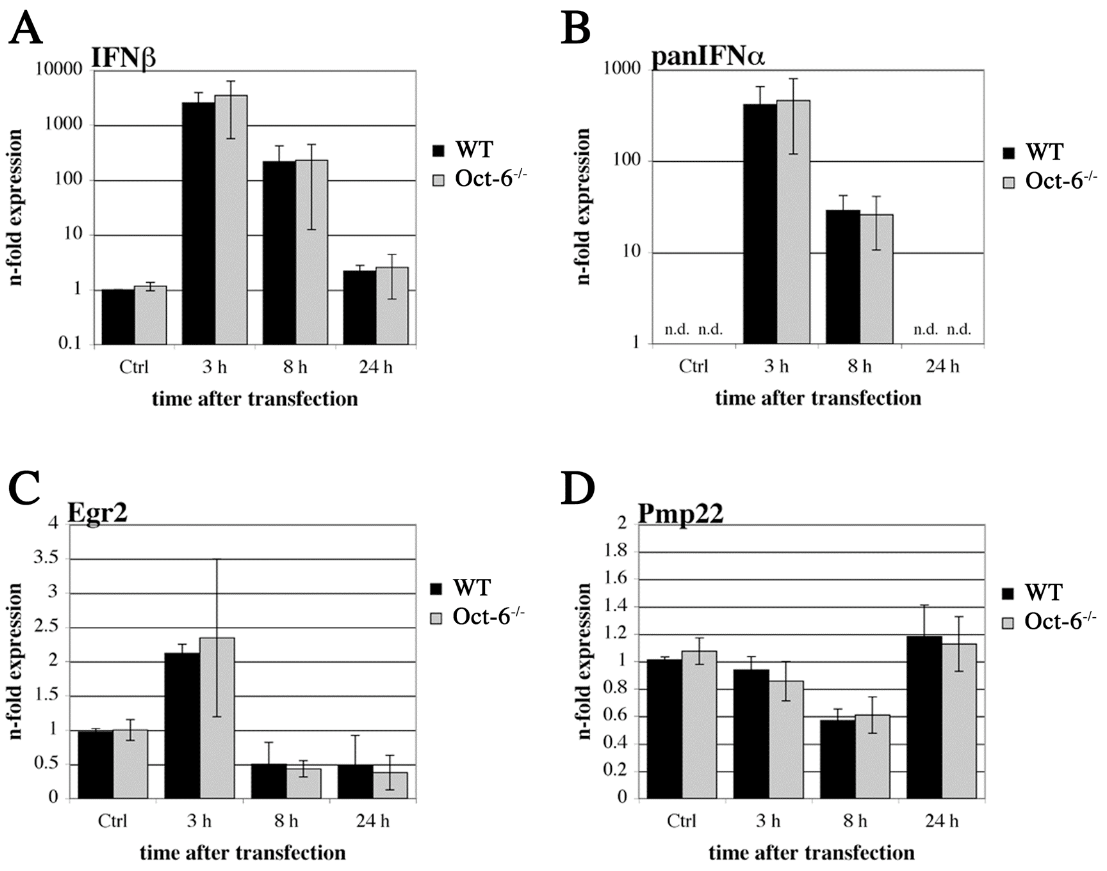

Additional file 5.

Absence of Oct-6 does not influence the expression patterns of panIFNα, IFNβ, Egr2 and Pmp22 upon GFP transfection in MEFs**.** WT and Oct-$6^{-/-}$ MEFs were transfected with an enhanced-GFP expression vector (GFP), or were left untransfected (Ctrl). mRNA levels of (A) IFNβ, (B) panIFNα, (C) Egr2 and (D) Pmp22 were determined by RT-qPCR at 3 h, 8 h, and 24 h after transfection. Ube2d2 was used as endogenous control. (A, C, D) Data are depicted relative to the WT Ctrl. (B) panIFNα could not be detected reliably in untreated cells (n.d.) and thus data normalised to the endogenous control are depicted (not additionally calibrated to untreated cells). Mean values ± SD from 3 independent experiments are shown.
