# Supplementary material for: Octamer-binding factor 6 (Oct-6/Pou3f1) is induced by interferon and contributes to dsRNA-mediated transcriptional responses
Source: BMC Cell Biol. 2010 Aug 5;11:61. doi: 10.1186/1471-2121-11-61 (PMC2924845; doi:10.1186/1471-2121-11-61)
Supplement: Additional file 6 — Absence of Oct-6 in macrophages has no impact on MCMV replication at a MOI of 0.1. Comparison of MCMV replication in WT and Oct-6-deficient foetal liver-derived macrophages at a lower MOI. [file 1471-2121-11-61-S6.PDF]

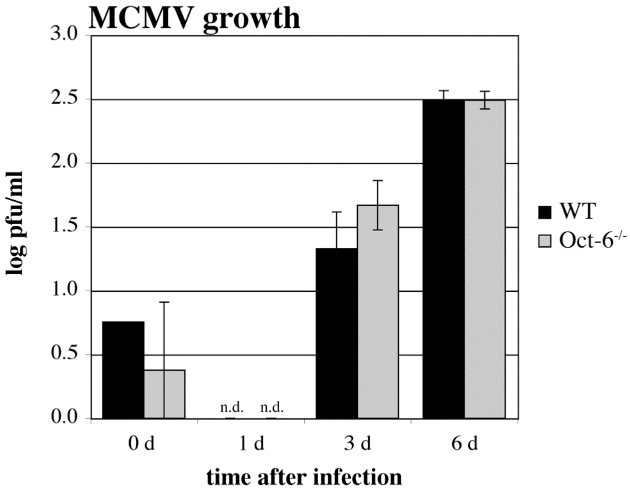

Additional file 6.

Absence of Oct-6 in macrophages has no impact on MCMV replication. WT and Oct-6$^{-/-}$ FLMs were infected with MCMV (MOI=0.1) for 90 min, washed with PBS and fresh medium was added. Supernatants were collected 0, 1, 3 and 6 days (d) after infection and virus titers were determined by plaque forming assays using Stat1$^{-/-}$ MEFs. Mean values ± SD of two experiments are shown.
