# Supplementary material for: Octamer-binding factor 6 (Oct-6/Pou3f1) is induced by interferon and contributes to dsRNA-mediated transcriptional responses
Source: BMC Cell Biol. 2010 Aug 5;11:61. doi: 10.1186/1471-2121-11-61 (PMC2924845; doi:10.1186/1471-2121-11-61)
Supplement: Additional file 7 — Differentially expressed genes as determined by microarray analysis that could be functionally annotated (96 out of 200; WT vs. Oct-6-/-, at least 2-fold difference, p <0.05). [file 1471-2121-11-61-S7.DOC]

**Supplemental table 1.** Genes differentially expressed between WT and Oct-6^-/-^ FLMs after poly(I:C) treatment.

| **Probe ID** | **FC** | ***p-value*** | **Gene Symbol** | **RefSeq** | **Description** |
| --- | --- | --- | --- | --- | --- |
| **Regulation of transcription (GO-BP GO:0045449)** | | | | | |
| 876633 | 0.3 | *0.0075* | *Ctnnd2* | NM_008729.1 | catenin (cadherin associated protein), delta 2 |
| 834086 | 0.3 | *0.0044* | *Rcor3* | NM_144814.2 | REST corepressor 3 |
| 515044 | 0.3 | *0.0101* | *Zfp691* | NM_183140.1 | zinc finger protein 691 |
| 452181 | 0.3 | *0.0433* | *Zmiz1* | NM_175264.2 | zinc finger, MIZ-type containing 1 |
| 553608 | 0.3 | *0.0014* | *Gtf2f2* | NM_026816.3 | general transcription factor IIF, polypeptide 2 |
| 315360 | 0.3 | *0.0201* | *Zfp775* | NM_173429.1 | zinc finger protein 775 |
| 784635 | 0.3 | *0.0077* | *9630041N07Rik* | NM_173387.1 | RIKEN cDNA 9630041N07 gene |
| 339824 | 0.4 | *0.0122* | *Egr3* | NM_018781.1 | early growth response 3 |
| 360325 | 0.4 | *0.0182* | *Foxk2* | NM_001080932.2 | forkhead box K2 |
| 675560 | 0.4 | *0.0480* | *Zfp414* | NM_026712.1 | zinc finger protein 414 |
| 883636 | 0.5 | *0.0356* | *Btg1* | NM_007569.2 | B-cell translocation gene 1, anti-proliferative |
| 388184 | 2.3 | *0.0176* | *8430426H19Rik* | NM_178875.4 | RIKEN cDNA 8430426H19 gene |
| 674148 | 2.9 | *0.0017* | *Snx13* | NM_001014973.2 | sorting nexin 13 |
| 576988 | 3.2 | *0.0208* | *2810021J22Rik* | NM_172403.2 | RIKEN cDNA 2810021J22 gene |
| 906799 | 3.3 | *0.0034* | *E4f1* | NM_007893.1 | E4F transcription factor 1 |
| **RNA splicing (GO-BP GO:0008380)** | | | | | |
| 466728 | 0.4 | *0.0481* | *Sfrs14* | NM_172755.2 | splicing factor, arginine/serine-rich 14 |
| 782547 | 0.4 | *0.0024* | *Lsm10* | NM_138721.1 | U7 snRNP-specific Sm-like protein LSM10 |
| 685184 | 0.5 | *0.0099* | *Sf3a1* | NM_026175.5 | splicing factor 3a, subunit 1 |
| **Ubiquitin cycle (GO-BP GO:0006512)** | | | | | |
| 628730 | 0.3 | *0.0383* | *Rnf41* | NM_026259.2 | ring finger protein 41 |
| 643747 | 0.4 | *0.0294* | *Klhl12* | NM_153128.1 | kelch-like 12 (Drosophila) |
| 665024 | 0.5 | *0.0434* | *Znrf4* | NM_011483.1 | zinc and ring finger 4 |
| 381530 | 2.5 | *0.0337* | *Senp5* | NM_177103.4 | SUMO/sentrin specific peptidase 5 |
| 363922 | 2.9 | *0.0324* | *Yod1* | NM_178691.2 | YOD1 OTU deubiquitinating enzyme 1 homologue (S. cerevisiae) NA 9930028C20 gene |
| 308725 | 4.2 | *0.0152* | *Senp8* | NM_027838.2 | SUMO/sentrin specific peptidase 8 |
| **Telomere organization and biogenesis (GO-BP GO:0032200)** | | | | | |
| 687306 | 0.3 | *0.0088* | *Terc* | NR_001579.1 | telomerase RNA component |
| 705683 | 3.1 | *0.0034* | *Pot1b* | NM_028370.1 | protection of telomeres 1B |
| **Regulation of Ras protein signal transduction (GO-BP GO:0046578)** | | | | | |
| 509621 | 0.4 | *0.0077* | *Arhgef11* | NM_001003912.1 | Rho guanine nucleotide exchange factor (GEF) 11 |
| 600711 | 0.4 | *0.0249* | *Tbc1d16* | NM_172443.2 | TBC1 domain family, member 16 |
| 603800 | 0.4 | *0.0456* | *Arhgef16* | NM_001112744.1 | Rho guanine nucleotide exchange factor (GEF) 16 |
| 832842 | 2.6 | *0.0206* | *Arhgef4* | NM_183019.2 | Rho guanine nucleotide exchange factor 4 |
| **G-protein coupled receptor protein signaling pathway (GO-BP GO:0007186)** | | | | | |
| 461332 | 0.3 | *0.0160* | *Olfr1410* | NM_146491.1 | olfactory receptor 1410 |
| 498376 | 0.3 | *0.0336* | *Olfr982* | NM_146854.1 | olfactory receptor 982 |
| 538641 | 0.3 | *0.0485* | *Oprd1* | NM_013622.2 | opioid receptor, delta 1 |
| 900911 | 0.4 | *0.0150* | *Olfr1425|Olfr1426* | NM_001011853.1 | olfactory receptor 1425|olfactory receptor 1426 |
| 371452 | 0.4 | *0.0219* | *Olfr167* | NM_146935.1 | olfactory receptor 167 |
| 405218 | 0.4 | *0.0321* | *Cysltr2* | NM_133720.1 | cysteinyl leukotriene receptor 2 |
| 302319 | 0.4 | *0.0143* | *Pcsk1n* | NM_013892.2 | proprotein convertase subtilisin/kexin type 1 inhibitor |
| 576856 | 0.4 | *0.0138* | *Olfr1013* | NM_146762.1 | olfactory receptor 1013 |
| 498112 | 2.3 | *0.0316* | *Cacnb4* | NM_146123.1 | calcium channel, voltage-dependent, beta 4 subunit |
| 895890 | 2.5 | *0.0031* | *Olfr598|Olfr597* | NM_001011845.1 | olfactory receptor 598|olfactory receptor 597 |
| 496110 | 2.5 | *0.0169* | *Olfr310* | NM_001011520.1 | olfactory receptor 310 |
| 388075 | 2.8 | *0.0427* | *Adra2a* | NM_007417.4 | adrenergic receptor. alpha 2a |
| **Monovalent inorganic cation transport (GO-BP GO:0015672)** | | | | | |
| 455948 | 0.5 | *0.0299* | *Kcnj2* | NM_008425.2 | potassium inwardly-rectifying channel, subfamily J, member 2 |
| 620798 | 0.5 | *0.0405* | *Catsper3* | NM_029772.1 | cation channel, sperm associated 3 |
| 554608 | 0.5 | *0.0392* | *Slc8a1* | NM_011406.2 | solute carrier family 8 (sodium/calcium exchanger), member 1 |
| **Vitamin B6 biosynthetic process (GO-BP GO:0042819)** | | | | | |
| 480631 | 0.5 | *0.0473* | *Pdxk* | NM_172134.1 | pyridoxal (pyridoxine, vitamin B6) kinase |
| 533138 | 2.9 | *0.0472* | *Pnpo* | NM_134021.1 | pyridoxine 5'-phosphate oxidase |
| **Protein palmitoylation (GO-BP GO:0018345)** | | | | | |
| 617536 | 0.2 | *0.0124* | *Zdhhc3* | NM_026917.4 | zinc finger, DHHC domain containing 3 |
| 437482 | 0.4 | *0.0111* | *Zdhhc17* | NM_172554.1 | zinc finger, DHHC domain containing 17 |
| **Protein kinase activity (GO-MF GO:0004672)** | | | | | |
| 922907 | 0.3 | *0.0156* | *Map3k12* | NM_009582.2 | mitogen activated protein kinase kinase kinase 12 |
| 467664 | 0.3 | *0.0256* | *Epha10* | NM_177671.2 | Eph receptor A10 |
| 851957 | 0.4 | *0.0064* | *Stk40* | NM_028800.2 | serine/threonine kinase 40 |
| 627826 | 2.3 | *0.0465* | *Prkci* | NM_008857.2 | protein kinase C, iota |
| 530372 | 2.4 | *0.0141* | *Epha4* | NM_007936.2 | Eph receptor A4 |
| 837461 | 2.4 | *0.0336* | *Wnk1* | NM_198703.1 | WNK lysine deficient protein kinase 1 |
| 357720 | 2.9 | *0.0036* | *Phka1* | NM_008832.2 | phosphorylase kinase alpha 1 isoform 1 |
| 926998 | 3.3 | *0.0011* | *Rab38* | NM_028238.5 | Rab38, member of RAS oncogene family |
| 464717 | 4.6 | *0.0077* | *Dyrk1b* | NM_010092.1 | dual-specificity tyrosine-(Y)-phosphorylation regulated kinase 1b |
| **N-methyltransferase activity (GO-MF GO:000817)** | | | | | |
| 642032 | 2.9 | *0.0374* | *1190005F20Rik* | NM_026876.3 | RIKEN cDNA 1190005F20 gene |
| 375317 | 3.3 | *0.0017* | *Setmar* | NM_178391.2 | SET domain and mariner transposase fusion gene |
| **Zinc ion binding (GO-MF GO:0008270)** | | | | | |
| 632096 | 0.4 | *0.0318* | *4933422H20Rik* | NM_001033775.3 | RIKEN cDNA 4933422H20 gene |
| 789662 | 0.4 | *0.0232* | *2610507B11Rik* | NM_001002004.1 | RIKEN cDNA 2610507B11 gene |
| 871506 | 0.4 | *0.0258* | *Gne* | NM_015828.2 | glucosamine |
| 325670 | 0.4 | *0.0463* | *Zfp11* | NM_172462.1 | zinc finger protein 11 |
| 594289 | 2.1 | *0.0157* | *AU017455* | NM_001033215.2 | EST AU017455 |
| 375461 | 2.4 | *0.0077* | *2010315B03Rik* | XM_001472446.1 | RIKEN cDNA 2010315B03 gene |
| 887858 | 2.4 | *0.0067* | *Ppp1r10* | NM_175934.2 | protein phosphatase 1, regulatory subunit 10 |
| 721573 | 2.8 | *0.0009* | *Mpi* | NM_025837.2 | mannose phosphate isomerase |
| **ATPase activity (GO-MF GO:0016887)** | | | | | |
| 409901 | 0.5 | *0.0299* | *Vps4b* | NM_009190.2 | vacuolar protein sorting 4b (yeast) |
| 807992 | 2.2 | *0.0411* | *Ddx27* | NM_153065.1 | DEAD (Asp-Glu-Ala-Asp) box polypeptide 27 |
| 556097 | 2.4 | *0.0238* | *Abcd4* | NM_008992.1 | ATP-binding cassette, sub-family D (ALD), member 4 |
| **Mitochondrion (GO-CC GO:0005739)** | | | | | |
| 331903 | 0.4 | *0.0179* | *4931431F19Rik* | XM_133663.5 | RIKEN cDNA 4931431F19 gene |
| 606179 | 0.4 | *0.0038* | *Pars2* | NM_172272.1 | prolyl-tRNA synthetase (mitochondrial)(putative) |
| 892845 | 0.4 | *0.0327* | *Slc25a36* | NM_138756.2 | solute carrier family 25, member 36 |
| 663810 | 0.4 | *0.0190* | *Papd1* | NM_026157.1 | PAP associated domain containing 1 |
| 770438 | 0.5 | *0.0418* | *Slc25a23* | NM_025877.2 | solute carrier family 25 (mitochondrial carrier; phosphate carrier), member 23 |
| 744134 | 2.1 | *0.0170* | *Timm9* | NM_001024853.1 | translocase of inner mitochondrial membrane 9 homolog (yeast) |
| 626456 | 2.4 | *0.0017* | *Grpel1* | NM_024478.2 | GrpE-like 1, mitochondrial |
| **Endoplasmatic reticulum (GO-CC GO:0005783)** | | | | | |
| 460096 | 0.2 | *0.0295* | *Caml* | NM_007596.2 | calcium modulating ligand |
| 731197 | 0.3 | *0.0033* | *Rcn3* | NM_026555.1 | reticulocalbin 3, EF-hand calcium binding domain |
| 670264 | 0.4 | *0.0403* | *Arsg* | NM_028710.2 | arylsulfatase G |
| 720766 | 2.4 | *0.0126* | *Duoxa2* | NM_025777.1 | dual oxidase maturation factor 2 |
| **Integral to membrane (GO-CC GO:0016021)** | | | | | |
| 608851 | 0.2 | *0.0386* | *Tspan31* | NM_025982.4 | tetraspanin 31 |
| 360339 | 0.3 | *0.0464* | *Klra2* | NM_008462.4 | killer cell lectin-like receptor, subfamily A. member 2 |
| 852764 | 0.4 | *0.0497* | *Flrt3* | NM_178382.2 | fibronectin leucine rich transmembrane protein 3 |
| 925399 | 0.4 | *0.0239* | *Lect1* | NM_010701.1 | leukocyte cell derived chemotaxin 1 |
| 412730 | 0.4 | *0.0323* | *Tmem86b* | NM_023440.2 | transmembrane protein 86B |
| 552370 | 0.4 | *0.0162* | *Tmem183a* | NM_020588.2 | transmembrane protein 183A |
| 365471 | 0.4 | *0.0027* | *Col13a1* | NM_007731.1 | procollagen, type XIII, alpha 1 |
| 564703 | 0.5 | *0.0050* | *Cmtm7* | NM_133978.1 | CKLF-like MARVEL transmembrane domain containing 7 |
| 828936 | 0.5 | *0.0143* | *E030010A14Rik* | NM_183160.1 | RIKEN cDNA E030010A14 gene |
| 754664 | 2.4 | *0.0201* | *Tnfrsf17* | NM_011608.1 | tumor necrosis factor receptor superfamily, member 17 |
| 513587 | 2.5 | *0.0386* | *Cd276* | NM_133983.2 | CD276 antigen |
| 440799 | 2.7 | *0.0451* | *Slc2a1* | NM_011400.1 | solute carrier family 2 (facilitated glucose transporter), member 1 |
| 760102 | 3.4 | *0.0295* | *Sdc1* | NM_011519.1 | syndecan 1 |
| 779189 | 4.0 | *0.0169* | *Tmem104* | NM_001033393.1 | transmembrane protein 104 |

Differentially expressed genes as determined by microarray analysis that could be functionally annotated (96 out of 200; WT *vs.* Oct-6^-/-^ after poly(I:C) treatment, at least 2-fold difference, *p<0.05*). FC: fold change between WT and Oct-6^-/-^ FLMs after poly(I:C) treatment; within groups sorted by FC; GO-BP: GO biological process; GO-MF: GO molecular function; GO-CC: GO cellular compartment.
